# Use of Off-Label Drugs in COVID-19: Clinicians’ Perceptions Based on a Cross-Sectional Observational Study

**DOI:** 10.7759/cureus.41819

**Published:** 2023-07-13

**Authors:** Anubha Sagar, Taruna Sharma

**Affiliations:** 1 Department of Pharmacology, Himalayan Institute of Medical Sciences, Dehradun, IND

**Keywords:** off-label drugs, misinformation, prescription patterns, pandemic perceptions, covid-19 infection

## Abstract

Introduction

The absence of a common National Treatment Guideline during the second wave of the COVID-19 pandemic in India resulted in different treatment strategies, and the use of "off-label drugs" (OfLDs) was one of them.

Aims

This study aimed to assess the proportion of doctors who prescribed OfLDs, their perceived appropriateness, and the factors leading to their use.

Settings and design

This is an undergraduate student research project, in which a web-based cross-sectional survey was conducted on doctors who delivered care to COVID-19 patients during the second wave of the pandemic in Uttarakhand, India.

Materials and methods

The minimum sample size was 370 (for a 95% confidence level, an alpha error of 0.5, and a power of 80%). Data were collected electronically using a validated questionnaire after institutional ethical clearance and the participants' consent.

Statistical analysis

This is a descriptive-analytical study.

Results

We received 419 completed responses; all specialties had seen COVID-19 patients, and 91.4% (383) of the doctors had provided care to COVID-19 patients in some way or the other. About 90.7% (380) of the doctors used OfLDs; 62.5% (262) agreed that OfLDs were beneficial, and 78.9% (331) disagreed on universal steroid use. Only 34.1% (143) felt that using OfLDs was ethical. About 16.9% (71) of the doctors believed that alternative medicine was a useful treatment adjunct, and 20% (84) of doctors prescribed OfLDs under duress. About 21.2% (89) believed that Remdesivir was the main treatment for the disease, and 18.6% (78) believed that Tocilizumab was the main treatment for the disease. Personal experience, conviction, or advice from peers were among the various reasons that were put forward for using OfLDs.

Conclusions

The use of OfLDs during the COVID-19 pandemic in India was extensive. It was done sometimes under pressure and was largely based on confusion (multiplicity of guidelines, many times at variance with each other) as well as on a personal or low level of scientific evidence forwarded to support the use.

## Introduction

The second wave of the COVID-19 pandemic in India (from mid-March to the end of May 2021), saw a surge in the use of several off-label drugs (OfLDs), including antibiotics, anthelminthic, immunomodulatory, antiviral, anticoagulant, monoclonal antibody, steroids, and a variety of vitamin/mineral subgroups.

The term “off-label drug” has many definitions. Under pandemic conditions, the WHO defines it as a repurposed, unregistered, experimental, unproven, untested (in humans or animals), or a trial/investigational drug [1]. OfLDs are commonly used in oncology, pediatrics, and critical care [2,3].

Most OfLDs for COVID-19 lack a firm grade of recommendation in international guidelines, even now. The confusion compounded during the peak of the pandemic when some of these found a mention in certain official guidelines. The guidelines issued by the Government of Uttarakhand mentioned the use of OfLDs. All India Institute of Medical Sciences and the Indian Council of Medical Research jointly issued guidelines for the treatment of COVID-19 patients and also mentioned the use of off-label drugs [4,5].

Unlike the United Kingdom, where a clear point-of-care algorithm existed for the management and the choice of drugs, there were multiple state and national guidelines in India (as mentioned in the preceding paragraph), which had variations among them. This left the doctors in a difficult situation in the selection of best management strategies.

We thus wanted to get a view of the doctors’ perceptions about the role of OfLDs in the treatment of COVID-19 infection. A PubMed search could not retrieve any study (even at the writing of this article) about this gap in knowledge.

The aims and objectives of our study were to determine the proportion of doctors who prescribed OfLDs to treat COVID-19-infected patients, the proportion of doctors who believe that COVID-19-infected patients can be managed appropriately using OfLDs, and the factors that lead to the use of OfLDs in the treatment of COVID-19 infection.

## Materials and methods

Study design

A web-based cross-sectional survey was conducted on doctors (all specialties) who delivered care to COVID-19 patients during the second wave of the pandemic. The phrase "doctors who delivered care" includes doctors involved in providing direct clinical care and doctors not involved in direct clinical care but working in hospital administration or policy-making (indirect care).

Study setting and participants

The study sample was drawn from doctors (all of them who were considered eligible) working across various hospitals in Uttarakhand, India, between July and November 2021. Prior to the study, the questionnaire was processed for validation through a pilot study on 15 doctors (which included ease of readability, ease of understandability, and discrepancies among other things).

The Institutional Ethical Committee (IEC) provided the necessary ethical clearance (SRHU/HIMS/ETHICS/2021/88 dated September 20, 2021).

Data collection

The web-based questionnaire was in English. It was open for eight weeks and was delivered via “Survey Monkey” (an online survey platform), on social media platforms (like WhatsApp, email, or Twitter) using URL links. The survey moved to more respondents via the technique of snowballing. The faculty/mentor guided the undergraduate student in the development of the questionnaire. A mandatory electronic consent form for participation provided on the first page of the survey was followed by a 20-item questionnaire. The questionnaire included 19 closed-ended questions (multiple-choice questions and Likert scale-based questions) and one open-ended question. Some multiple-choice questions were allowed more than one answer. Five questions pertained to basic demographic data (gender, medical specialty, qualification level, professional level like trainee/specialist, and the number of COVID-19-infected patients treated), and 15 questions pertained to the perception and understanding of the use of OfLDs in the COVID-19 infection. The last question (optional, open-ended) had free-text responses. The OfLDs probed were Hydroxychloroquine, Doxycycline, Azithromycin, Vitamin C and Vitamin D, Zinc supplements, Ivermectin, and plasma therapy. Questions were framed separately for Remdesivir and Tocilizumab. Both of these drugs were not grouped with the other OfLDs as they received authorization for use in COVID-19 patients in certain scenarios.

Bias

The target of the survey was doctors from hospitals and medical colleges in the state. Since the survey moved by snowballing effect, it captured some responses from the primary care and private practice clinics. However, the survey results would be reflective of the opinion of secondary and tertiary care only and not of primary care. This is later revealed in Table 1 under the respondents' area of work. We used the electronic technique of dissemination, which meant that the survey had the potential to miss those doctors who are not on social media. Some doctors on social media might not have had the time to respond to the survey. We decided to accept this bias as it was difficult to organize a face-to-face collection of data during the pandemic.

**Table 1:** Basic demographic data of the respondents

Gender distribution of the respondents	
Female	114 (27.2%)
Male	303 (72.3%)
Prefers not to reveal	02 (0.5%)
Respondent’s area of work	
Hospital/Medical college	316 (75.4%)
Non-hospital setting [like a private clinic/telephonic advice/social media/teleconsultation etc.]	60 (14.3%)
Both of the above options	43 (10.3%)
Academic qualification of respondents	
Postgraduate (e.g., MD/MS/DM/MCh/Diploma/equivalent)	325 (77.6%)
Medical graduate only	94 (22.4%)
Total	419
Work nature profile	
Academic teaching roles	103 (24.6 %)
Specialist roles in hospital	93 (22.2%)
Various other appointments (e.g., training)	223 (53.2%)
Total	419
Specialty-wise distribution of the survey respondents	
Clinical Medicine and Allied: General Medicine and Subspecialty of Medicine	221 (52.7%)
Clinical Surgery and Allied: General Surgery and Subspecialty of Surgery	44 (10.5%)
Others, e.g.: laboratory sciences/radiological sciences/basic sciences	53 (12.6%)
Currently not a specialist	75 (17.9%)
Currently administrative appointments only and not in patients' work	26 (6.2%)
Total	419

Study size

About 10,000 doctors are registered with the Uttarakhand Medical Register (all are considered eligible). We used sample size calculation for a survey. The calculator is available on the website of Survey Monkey (a global website for conducting surveys). This provided a sample size of 370 doctors (with a 95% confidence level, a margin of error of 5%, and a power of 80%).

Statistical methods

The data was subjected to descriptive analysis and, where applicable, statistical methods. Simple proportions and percentages have been used for the analysis. Statistical analysis using SPSS (Statistical Package for the Social Sciences) version 25 (IBM Corp., Armonk, NY) was used to compare the significant differences in proportions (within subgroups using two-tailed Z score and the p-value) where applicable. The subgroups analyzed were clinical medicine/allied versus nonclinical specialties. A p-value less than 0.05 was taken as significant. The electronic format clearly flagged any incomplete document and excluded it from the analysis. The data collected was non-identifiable, thus anonymous and blinded.

## Results

A total of 466 responses were obtained, out of which 47 were incomplete. We thus had 419 completed responses, which were analyzed, delivering a survey completion rate of 90%. The average time taken to complete the survey was 5 minutes and 15 seconds. About 167 respondents chose to answer the optional question with free-text responses.

Table 1 shows the basic demographics of the respondents. About 91.4% (383) of doctors had delivered care to COVID-19 patients (N = 419). “Care of COVID-19 patients” included both direct clinical care (face-to-face and/or distance electronic consultations) and indirect care (through doctors in hospital administration and policy-making). Doctors in direct clinical care had a workload ranging from less than two to more than 10 patients per week. About 8.6% (36) of doctors had not managed any COVID-19 patients. These doctors had provided neither direct nor indirect care to the COVID-19 patients. OfLDs were prescribed by 90.7% (380) of doctors.

For the purpose of analyzing questions based on the Likert scale, responses marked as "agree" or "strongly agree" were clubbed together as effectively meaning "agree." Similarly, "disagree" and "strongly disagree" were effectively clubbed as "disagree."

Over 50% (222) of doctors affirmed the use of Remdesivir, and around 40% (169) affirmed the use of Tocilizumab (N = 419). About 62.5% (262) of respondents agreed that OfLDs (except Remdesivir and Tocilizumab) were beneficial for COVID-19 infection. However, 28.6% (120) of doctors agreed that the use of OfLDs was based on strong scientific evidence. About 21.2% (89) of doctors agreed that Remdesivir was the main lifesaving drug in the management of COVID-19 infection, whereas 18.6% (78) of doctors deemed Tocilizumab as the most important lifesaving drug.

About 78.9% (331) of doctors disagreed that steroids should be given to all patients with COVID-19 infection, and 16.9% (71) of doctors agreed that complementary alternative medicine (CAM) has a beneficial role in the management of COVID-19 infection.

**Figure 1:**
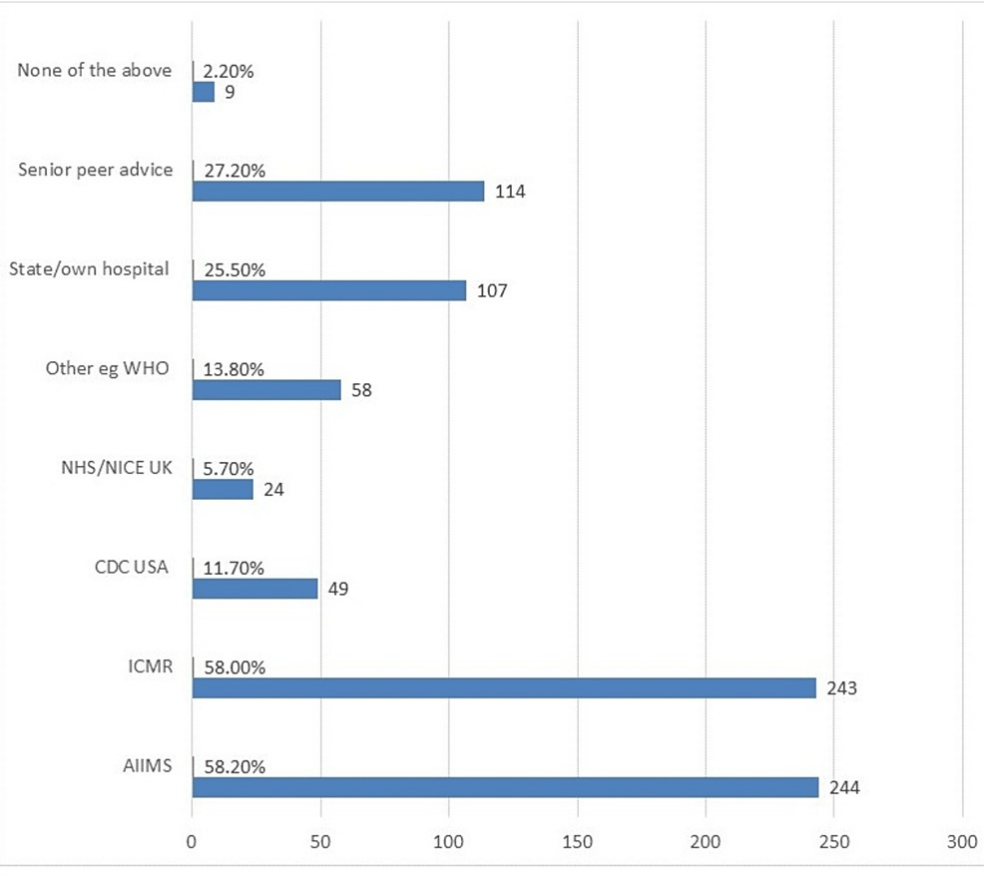
Clinical decision-making tools used (multiple options allowed), N = 419 NHS: National Health Service; NICE: National Institute for Health and Care Excellence; ICMR: Indian Council of Medical Research; AIIMS: All India Institute of Medical Sciences.

Only 34.1% (143) of doctors agreed that OfLD use is ethical in the management of COVID-19 infection. The rest were undecided. About 20% (84) of respondents admitted to prescribing OfLDs under patient or peer pressure, while 64% (268) of doctors clearly responded as not having succumbed to the same, and 16% (67) of doctors either were unsure or had not faced such a situation. Figure 1 displays the decision-making tools used for the management of COVID-19 (more than one choice was permitted). Free-text responses on the reasons why the doctors used OfLDs are depicted in Table 2.

**Table 2:** Free-text responses on the reasons why doctors used off-label drugs in COVID-19 ICMR: Indian Council of Medical Research; AIIMS: All India Institute of Medical Sciences.

Type of medicine	Examples of responses
Propaganda-based medicine	Didn’t stop vitamins from being taken as per social media/TV advice; vitamins boost immunity and are harmless; some antibiotics and Ivermectin seem fairly effective in early symptoms or mild symptoms.
Eminence-based medicine (seniority and white-haired, balding physician-based medicine)	Took the senior physician’s advice; personal experience; against my principles and hence not used them.
Confidence-based medicine	Ivermectin: felt strongly about effectiveness; some antibiotics and steroids: extremely effective in prophylaxis and early management. Most Indians are vitamin- and Zinc-deficient; use is justified in any viral illness, not only COVID-19; own critical appraisal of published evidence.
Confusion-based medicine	Evidence evolves every day, and findings are confounding. We need to “Do No Harm,” just to give something than giving nothing.
Vehemence-based medicine (substitution of the volume of voice for evidence)	Vitamin D has proven its usefulness in COVID-19. My father improved with Tofacitinib.
Eloquence-based medicine (sophistication substituting for science)	Evidence is nice, but common sense is better; out-of-box thinking and not expecting "others" from the West to provide us with guidelines.
Diffidence-based medicine (shyness from lack of confidence)	You never know what might work, not much to offer. Patients’ expectations to save them by whatever available means placebo effect.
Nervousness-based medicine (scared of litigation)	Pressure from relatives; some had powerful social status. Trend and pressure: Fabiflu and Remdesivir.
Enforcement-based medicine (mandatory guidance, whether you agree or not)	Had no option but followed the Indian government/AIIMS/ICMR guidelines.

We applied filters and analyzed clinical medicine/allied as one group and the rest as non-medical specialties (for the breakdown of numbers, see Table 1 under the specialty-wise distribution of the survey respondents). There was no significant difference between the two groups on the perceptions of the usefulness of OfLDs, whether their use is based on scientific evidence, views on Remdesivir or Tocilizumab, or if the use of OfLDs is ethical. Two-tailed Z scores and p-values were calculated. We excluded the group of doctors who identified as “currently not a specialist” (likely those in training and interns) in either group. The groups were similar as they were randomly selected by snowballing, differing only in specialty as clinical medicine specialties and allied versus nonclinical specialties and allied. The subgroups were not powered for analysis. At the same time, there was no data to indicate a difference between the two groups.

## Discussion

Our survey provided 419 responses, which is beyond the sample size of 370. Around 91.4% (383) of doctors had provided care to COVID-19 patients (including non-medical/allied specialties), mainly due to reallocation to medical wards, a finding that was consistent globally. The survey was conducted via the technique of snowballing, so it also reached 8.6% (36) of doctors who had not provided care in any form to COVID-19 patients. The sample size was adequate even without including their responses. We could have excluded their responses, but it was decided by the authors to include their responses since they were doctors who have a basic knowledge of drugs and the disease. These doctors did not provide care at the time when we conducted the survey but have the potential to do so in the future. Hence, their views mattered, and their responses were also analyzed.

In our study, 27.2% (114) of the doctors were female compared to 16.8% nationwide and 11.1% in the state of Uttarakhand [6]. Gender-based comparison of responses was not attempted as it was not expected to be clinically relevant. Over 77.6% (325) of doctors had a postgraduate degree or higher. The survey may be somewhat skewed toward perceptions of doctors in tertiary care as it was sent to hospitals. This bias was accepted during the design of the study as explained in the methods earlier.

The pandemic brought telemedicine to the forefront for technologically enabled doctors and patients [7]. About 25% (105) of doctors did electronic consultations for COVID-19 patients irrespective of specialty or qualification. Over 90% of doctors used OfLDs for the management of COVID-19 patients. This was similar to how it was done elsewhere in the world [8]. About 16.9% (71) of doctors agreed that CAM has a beneficial role in COVID-19 infection, which is a matter of some concern because they were allopathic doctors who were not supposed to be prescribing CAMs. Second, CAMs have no established role in most diseases we treat including COVID. Third, CAMs are different from OfLDs. OfLDs are repurposed drugs that have been otherwise scientifically researched; however, CAMs have not. Around 62.5% (262) of doctors agreed that OfLDs were beneficial in the treatment of COVID-19 patients. However, only 28.6% (120) believed that the use of OfLDs had a strong scientific basis (a bit of a worry as elaborated in free-text responses).

Except for low molecular weight heparin and steroids, none of the OfLDs had a strong scientific basis for usage in COVID-19. The remaining OfLDs either had no scientific basis or had very limited and conditional recommendations. Hence, it is concerning that more than a quarter of doctors believed that OfLDs had a strong scientific basis for use. They provided personal reasons supporting their belief in the free-text responses. The majority of doctors accepted that the evidence for the use of OfLDs was not strong. However, they accepted the use as the need of the hour demanded them. This was evident from their rationalization as brought out in the free-text responses. Thus, while the doctors accepted the use of OfLDs based on limited knowledge, most were aware that the use was not based on strong evidence. This is a positive aspect, which informs us that the medical fraternity did not lose its wisdom even at the peak of the stress.

Despite the fact that these drugs had a conditional and very specific recommendation, 18.6% (78) of doctors believed that Tocilizumab and 21.2% (89) of doctors believed that Remdesivir were the main lifesaving drugs in the management of COVID-19 infection [9]. In severe COVID-19 infection, the use of Remdesivir (weak recommendation) was permitted for a subset of cases requiring oxygen supplementation (non-invasive mechanical ventilation or a high-flow device) [9,10]. Humanized monoclonal antibody Tocilizumab with dexamethasone (weak recommendation) was approved for patients going into rapid respiratory decompensation. FDA had approved only Remdesivir for the treatment of COVID-19 infection during the second wave of the COVID-19 pandemic in India [10].

Dexamethasone had a Grade-A recommendation for use in patients requiring oxygen therapy [4,11]. In our study, 78.9% (331) of doctors disagreed that steroids are required for all patients, although we expected this figure to be 100%. However, it was comforting that the majority of doctors were scientifically correct. A common perception (from social media) that most patients were being offered steroids was at variance from our finding, maybe because our study did not include primary care.

About 34.1% (143) of doctors felt that the use of OfLDs in COVID-19 treatment was not ethical, while the rest were undecided. FDA (in 2009), formalized an existing “expanded access pathway” enabling seriously ill patients (not qualifying for a trial) to access OfLDs [2]. In 2016, WHO issued similar guidance for infectious disease outbreaks but required fulfillment of certain conditions [1]. The practice of OfLD use is neither encouraged nor prohibited by the regulatory agencies. The US FDA allows doctors to use legally approved medicines prescribed outside the officially permitted indications, but the patient needs to be informed [12]. Europe is more conservative, with some countries having specifically made legislation to limit the use or created regulatory frameworks around OfLD use [13]. Patients can demand the correct treatment (mostly on the label). However, the prescriber is responsible for prescribing the off-label treatment when an on-label drug is not available. When off-label treatment is not available, the patient retains the right to on-label treatment. The fundamental idea remains that OfLD prescription remains the responsibility of the prescriber, and the patient’s right to request on-label treatments remains if off-label treatment is not appropriate. The most common subcategories for off-label use are modification of doses, route of administration, a different indication, formulation, or age groups. This is a learning opportunity for the doctors, given the ethical and legal framework that OfLDs use as discussed earlier.

About 20% (84) of doctors admitted to prescribing medicines under peer pressure or on patients’ or relatives’ request. The law, violence against doctors, the out-of-pocket cost of medical treatment (under loans/mortgages), and professional indemnity need to be borne in mind before blaming the doctors for prescribing off-label treatment in such cases.

Grade-A recommendation exists for the use of anticoagulant prophylaxis in hospitalized COVID-19 patients; however, convalescent plasma and routine antibiotics are not recommended [11,14,15]. This was not followed by doctors in our study, mainly because of different guidelines adhered to (Figure 1). Various types of medicine other than evidence-based medicine were also followed (Table 2) [16]. Multiple guidelines (national/state/institutes of repute) existed in India during the second wave of the pandemic, with no agreed convergence on which guidelines to follow. This left the doctors to fend for themselves. These guidelines were at variance with each other, in contrast to the United Kingdom where every hospital followed only one treatment protocol, i.e., the UK National Institute for Health and Care Excellence (NICE) guidelines [1,2,17]. Uniform guidelines provide safe protocols including hospital transfers, curbing of misinformation, and legal safety for the medical staff.

The free-text responses provided us with a lot of insight into the reasons for the use of OfLDs. Whatever the reasons, the authors believe that the medical fraternity was equally confused with the barrage of new information on the disease. Evolving international evidence and a scarcity of time to perform an appraisal of the evidence on an individual level were some of the reasons cited for off-label use. Another factor was the presence of a multitude of local guidelines, which caused confusion among doctors. The fear of causing harm to the patients by withholding the use of drugs being prescribed by other doctors was another reason for off-label drug use. Doctors wished well for the patients while being relatively safe and possibly beneficial. Thus without cognitive resonance, the doctors were following the principles of the US FDA and the WHO on the use of OfLDs. We need to focus on evolving single national guidelines for common illnesses. Implementation, adherence, and periodic audit on their use need to be carried out. The guidelines also need to be regularly updated as per new evidence available. This will prevent confusion similar to what happened during the COVID-19 pandemic.

A study by Dasgupta found that the use of OfLDs in COVID-19 treatment resulted in more fulminant COVID-19 manifestations in some cases, concluding that increasing pharmacovigilance in such settings is important [18].

Anyone can succumb to misinformation and half-truths. An overwhelming scale of multiplication happens to misinformation and pseudoscience on social media. This misinformation bears a strong chance of acceptance, and it cannot be undone by retracting this information once accepted. Thus, the contact with half-truths continues (e.g., the continued use of hydroxychloroquine in spite of scientific discredit) [19]. Assisted decision-making (between doctors and patients) leads to better patient acceptance, with patient autonomy being central [20].

Our study has strengths and limitations. Our sample was statistically adequate with a good breadth of specialties; however, it was drawn largely from the tertiary care setting, so it did not capture data from the primary care setting. Second, it is the only study of its kind on the subject, to the best of our knowledge. Third, being an internet and social media-driven survey, the attention span of users is small, the distraction rate is high, and the time available to doctors based on their busy schedules is limited. As a result, not everyone who intended to fill out the survey might have done it. An unavoidable sampling bias may have crept in. Fourth, we received 47 incomplete responses from willing respondents, which could not be analyzed. A completion rate of 80%-85% is considered good for internet surveys. Our completion rate was 90%. Finally, we would be cautious in the generalization of the findings because the study was unable to capture data from the primary care setting. However, the authors feel that for secondary and tertiary care, the findings may be appropriate (with a caveat that it was targeted electronically and not through a random sampling technique).

## Conclusions

A glimpse into the perceptions of doctors on the use of OfLDs during the pandemic in India revealed that the use of OfLDs was extensive and sometimes under pressure. It was largely based on confusion due to the multiplicity of guidelines used in addition to personal or low level of scientific evidence forwarded to support the use.
